# Predicting preoperative muscle invasion status for bladder cancer using computed tomography-based radiomics nomogram

**DOI:** 10.1186/s12880-024-01276-7

**Published:** 2024-04-27

**Authors:** Rui Zhang, Shijun Jia, Linhan Zhai, Feng Wu, Shuang Zhang, Feng Li

**Affiliations:** Department of Radiology, Xiangyang Central Hospital, Affiliated Hospital of Hubei University of Arts and Science, Xiangyang, 441021 Hubei China

**Keywords:** Bladder cancer, Computed tomography, Radiomics, Muscle invasion, Nomogram

## Abstract

**Objectives:**

The aim of the study is to assess the efficacy of the established computed tomography (CT)-based radiomics nomogram combined with radiomics and clinical features for predicting muscle invasion status in bladder cancer (BCa).

**Methods:**

A retrospective analysis was conducted using data from patients who underwent CT urography at our institution between May 2018 and April 2023 with urothelial carcinoma of the bladder confirmed by postoperative histology. There were 196 patients enrolled in all, and each was randomized at random to either the training cohort (*n* = 137) or the test cohort (*n* = 59). Eight hundred fifty-one radiomics features in all were retrieved. For feature selection, the significance test and least absolute shrinkage and selection operator (LASSO) approaches were utilized. Subsequently, the radiomics score (Radscore) was obtained by applying linear weighting based on the selected features. The clinical and radiomics model, as well as radiomics-clinical nomogram were all established using logistic regression. Three models were evaluated using analysis of the receiver operating characteristic curve. An area under the curve (AUC) and 95% confidence intervals (CI) as well as specificity, sensitivity, accuracy, negative predictive value, and positive predictive value were included in the analysis. Radiomics-clinical nomogram’s performance was assessed based on discrimination, calibration, and clinical utility.

**Results:**

After obtaining 851 radiomics features, 12 features were ultimately selected. Histopathological grading and tortuous blood vessels were included in the clinical model. The Radscore and clinical histopathology grading were among the final predictors in the unique nomogram. The three models had an AUC of 0.811 (95% CI, 0.742–0.880), 0.845 (95% CI, 0.781–0.908), and 0.896 (95% CI, 0.846–0.947) in the training cohort and in the test cohort they were 0.808 (95% CI, 0.703–0.913), 0.847 (95% CI, 0.739–0.954), and 0.887 (95% CI, 0.803–0.971). According to the DeLong test, the radiomics-clinical nomogram’s AUC in the training cohort substantially differed from that of the clinical model (AUC: 0.896 versus 0.845, *p* = 0.015) and the radiomics model (AUC: 0.896 versus 0.811, *p* = 0.002). The Delong test in the test cohort revealed no significant difference among the three models.

**Conclusions:**

CT-based radiomics-clinical nomogram can be a useful tool for quantitatively predicting the status of muscle invasion in BCa.

**Supplementary Information:**

The online version contains supplementary material available at 10.1186/s12880-024-01276-7.

## Introduction

One of the most prevalent urological malignancies and the tenth most common malignancy worldwide is bladder cancer (BCa) [1]. The most prevalent histological type of BCa is urothelial carcinoma [2]. Based on the degree of tumor infiltration into the bladder wall, BCa can be classified pathologically as either muscle invasive bladder cancer (MIBC) or non-MIBC (NMIBC) [2, 3]. Transurethral cystectomy for bladder tumor (TURBT) is the recommended course of treatment for NMIBC, whereas MIBC typically necessitates additional therapies such radical cystectomy, adjuvant chemotherapy, and radiotherapy [3]. This suggests that one of the most significant criteria determining therapy choices for the clinical management of BCa [4, 5] is histological muscle invasion.

Currently, cystoscopic biopsy is the main approach for tumor diagnosis and clinical staging [4, 6]. However, accurate preoperative diagnosis of muscle invasiveness is not an easy task. Since biopsies are operator-dependent, incorrect staging may occur due to inadequate biopsy samples or problems with sample quality [6, 7]. According to previous studies, 20–80% of BCa patients are incorrectly staged due to differences in biopsy [8, 9].

The most frequent noninvasive evaluation technique in BCa patients is computed tomography (CT), which can be used to locate the tumor and assess the tumor’s quantity, dimension, relationship to surrounding tissues, and metastasis [9]. However, traditional CT scans cannot be used to evaluate muscle invasion in BCa because they have poor soft-tissue resolution and cannot distinguish between the different layers of the bladder wall. Magnetic resonance (MR) is more accurate than CT, but the high cost, prolonged scanning time, and numerous contraindications of MR prevent it from being used widely [9, 10]. In addition, imaging remains a subjective assessment process based on the radiologist’s experience. Therefore, there is a need to develop more accurate techniques to assess BCa invasiveness.

Radiomics is a new imaging analytical tool that quantifies medical image data to provide information on the morphological features, size, and location of lesions, allowing physicians to make more precise diagnoses, particularly in oncology [5–7]. Computers extract potentially important information from large amounts of medical image data and use different algorithms to build models to explain the association between images and diseases [11]. These models can help physicians detect tumor lesions and assess the aggressiveness of the lesions, and estimate the prognosis and response to treatment in cancer patients [11, 12]. Previous studies have demonstrated the high diagnostic performance of multi-sequence MR-based radiomics in predicting muscle invasion in BCa [5, 6, 9]. A radiomics analysis conducted by Zhang et al. using CT-enhanced images from 441 BCa patients revealed the better diagnostic performance of the radiomics model in assessing muscle invasion of BCa [7].

Therefore, to guide the choice of therapeutic treatment option, this study aimed to assess the viability of a radiomics-clinical nomogram for determining the muscle invasion status of BCa.

## Materials and methods

### Patients

The Xiangyang City Centre Hospital’s institutional review board approved this retrospective investigation and removed the demand for informed consent.

**Fig. 1:**
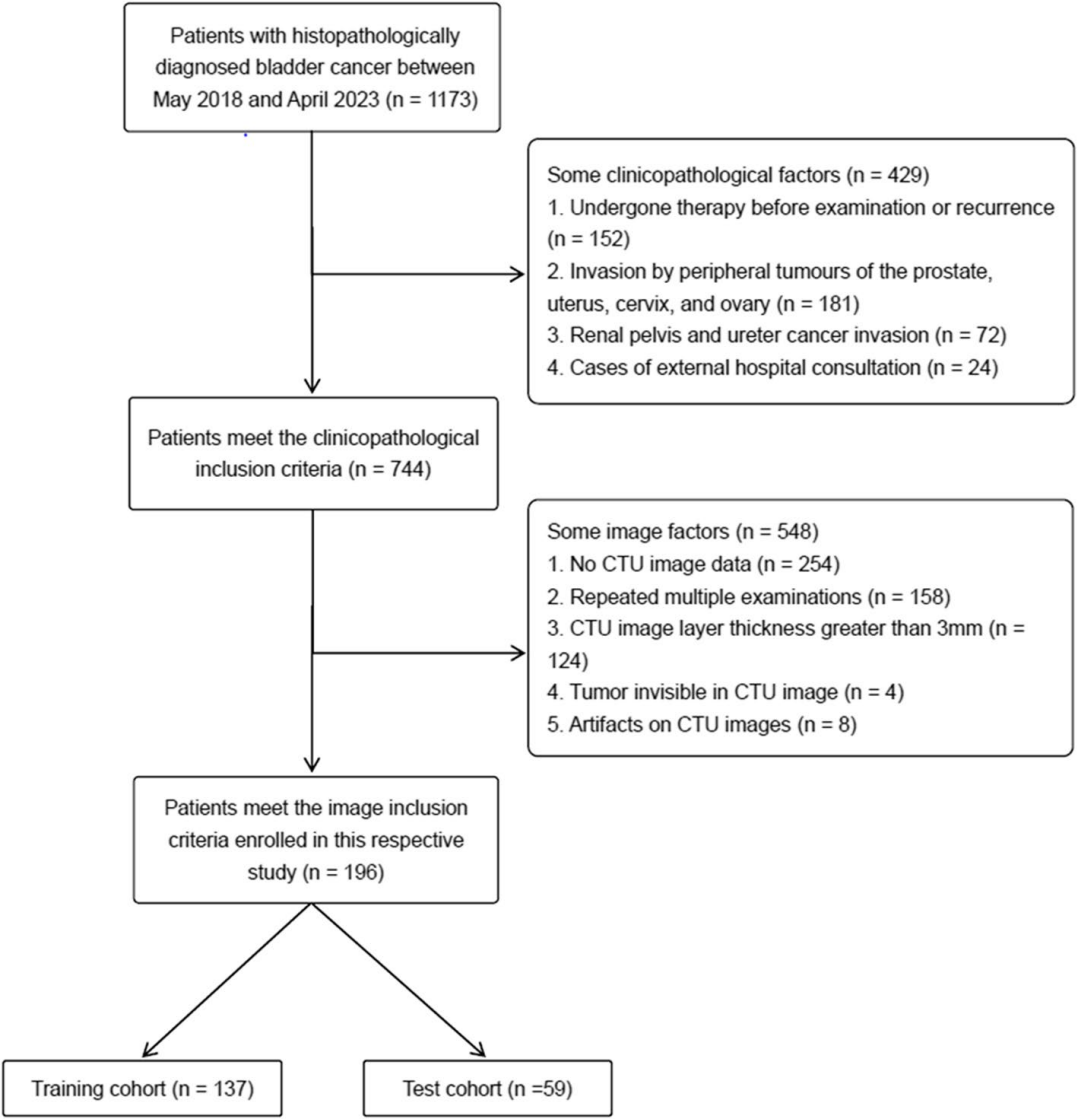
Patient selection flowchart illustration

We searched our hospital database for patients with BCa confirmed by postoperative pathology from May 2018 to April 2023. These criteria were used to determine inclusion: [1] patients who underwent TURBT or radical cystectomy with pathologically confirmed urothelial carcinoma, and [2] patients who underwent CT urography (CTU) within the preoperative period. The following criteria were used to exclude patients: [1] artifacts on CTU images, [2] incomplete image sequence and/or layer thickness greater than 3 mm, [3] lesion width lower than 5 mm, [4] underdistended bladder, [5] biopsy or treatment such as chemotherapy or radiotherapy prior to the CT examination, and [6] muscle layer undistinguishable in specimens resected by TURBT. A total of 196 patients were included, including 97 NMIBC and 89 MIBC cases, as shown in Fig. 1. Cases were divided into training and test cohorts (*n* = 137, *n* = 59) by 7:3 stratified random sampling.

The patient’s demographic and clinicopathological information was retrieved from their medical record, including age, sex, history of smoking, clinical complaint (e.g., hematuria, hydronephrosis, or incidental findings), infiltrative status of the bladder wall’s muscular layer, and histopathological grading (high and low grades as determined by using biopsy prior to surgery). Moreover, imaging information was retrieved from the patient’s CT images, and the number of lesions, calcifications, lesion length and tortuous blood vessels visible to the naked eye around or within the lesions were documented (Supplementary Material S1).

### CT data and image acquisition

All enrolled patients underwent CTU within 1–2 weeks prior surgery. Before the scan, patients were instructed to drink between 800 and 1000 ml of water, but not to urinate until the scan was over. After scanning, 50 mL of ioversol or 100 mL of iopamidol were intravenously administered, followed by 50 mL of saline at a rate of 3 mL/s. Images of the renal corticomedullary, nephrographic, and excretory phase were obtained at 25 s, 75 s, and 300 s after the thresholding of the thoracoabdominal aortic junction was reached. Subsequent analyses used only axial nephrographic phase images. Multidetector CT scanners with 64 to 128 detector rows (Siemens Healthineers, Philips Brilliance, and Philips Brilliance iCT) were used to obtain CT images. The scanning parameters were 120 kV, automatic mA settings (range 80–320 mA), layer spacing of 1 mm, and layer thickness of 1.5–3 mm. Soft tissue algorithm (window width (WW): 300–500 HU, window level (WL): 45–60 HU) were used after imaging.

### CT image segmentation and feature extraction

Three-dimensional regions of interest (3D-ROIs) were manually outlined on thin-layer CT images during the nephrography phase using the ITK-SNAP program (version 4.0.1; http://itk-snap.org). Only the largest lesion in patients who had multiple lesions was chosen for segmentation in this study. 3D-ROIs along the tumor margins were manually drawn by radiologist 1 (ZR, with 6 years of genitourinary imaging experience and 5 years of tumor segmentation experience). Radiologist 1 was uninformed of the status of muscle invasion in its postoperative pathology. To ensure the reproducibility of the regions of interest (ROI), intragroup correlation (ICC) was used to assess intra-observer agreement. We randomly selected 30 patients, and the ROI was manually outlined again after 4 weeks by the same radiologist and another one (ZLH, with 3 years of experience in genitourinary imaging and tumor segmentation). A good agreement was defined as an ICC of 0.75 or higher.

The PyRadiomics package (version 3.0.1, available at https://github.com/AIM-Harvard/pyradiomics.git) and Python (version 3.7.5) were used to obtain the radiomics features from the CT images. The original and wavelet-filtered image allowed for the retrieval of all radiomics features, which could be divided into seven groups, namely first-order statistics, shape features, glcm features, gldm features, glrlm features, glszm features and ngtdm features. The features extraction method is available from https://pyradiomics.readthedocs.io/en/latest. Finally, 851 features were extracted in each volume of interest of CT images, respectively.

### Feature selection and Radiomics model building

All features were normalized using z-score normalization prior to feature selection and model development. The ICC was used to assess the repeatability of each radiomics feature both intra- and inter-observer. In our study, only the features with ICC values greater than 0.75 were included. Features filtering was performed by using a significance test (Student’s t-test if the data adhered to a normal distribution, otherwise the Mann-Whitney U test) to select the features with high predictive power (*p* < 0.05). The most beneficial predictive muscle invasion status-related features were then chosen from the training cohort using the least absolute shrinkage and selection operator (LASSO) and 10-fold cross-validations. The radiomics score (Radscore) was obtained by applying linear weighting based on the selected features by the LASSO algorithm.

### Development and performance of a Radiomics-clinical nomogram

The included clinical indicators were subjected to independent univariate and multivariate analyses to identify the clinicopathological predictors for muscle invasion and create a clinical model. The final predictors for muscle invasion were chosen by using a multivariate logistic regression analysis that included the Radscores and the independent clinical indicators. The approach used forward stepdown selection with a liberal *p* < 0.05 as the retention criteria. Based on the results of the multivariate logistic regression analysis, a radiomics-clinical nomogram was created. Utilizing the Hosmer-Lemeshow test and the calibration curve, the radiomics-clinical nomogram’s calibration was evaluated.

### Models comparison and clinical usefulness evaluation

To further assess the applicability of the radiomics-clinical nomogram, it was compared with the clinical and the radiomics model. The diagnostic efficacy of the different models for BCa prediction was assessed by using receiver operating characteristic (ROC) curve to quantify the assessment power of each model. The area under the ROC curve (AUC) and 95% confidence intervals (CI) along with specificity, sensitivity, accuracy, negative predictive value (NPV), positive predictive value (PPV) were used to analyze the diagnostic efficacy of the radiomics models. Decision curve analysis (DCA), which calculated the net benefit at different threshold probabilities and compared the radiomics-clinical nomogram with the clinical model, was used to evaluate the clinical value of the radiomics-clinical nomogram.

### Statistics

The statistical analysis was completed using R (version 3.6.1, accessible at https://www.r-project.org). Image production was performed using Microsoft Visio for Windows (version 2021). Data that failed to conform to the normal distribution criteria were compared between the two groups using the Mann-Whitney U test. Following a normality check, the continuous data were used the Student’s t-test. Normal data were represented as mean ± standard deviation. Counting data were reported as the number of cases (rate), and the chi-square test was applied to compare the two groups. Inter-observer reproducibility of radiomics characteristics was assessed using the ICC to evaluate inter-observer agreement between radiologists, with coefficients greater than 0.75 indicating good reproducibility. The diagnostic efficacy of the clinical and radiomics model, as well as radiomics-clinical nomogram for BCa prediction were assessed using ROC curve. To determine the *p* value for the AUC, DeLong’s test was utilized. The two-sided p value threshold for statistical significance was 0.05.

## Results

### Clinical characteristics

In this study, data were collected from 196 patients. The test cohort consisted of 59 patients and the training cohort of 137 patients. Age, sex, history of smoking, number of lesions, clinical symptoms, histopathological grading, calcification, lesion length, and presence of tortuous blood vessels were among the clinical traits present in both the training and test cohorts. The training and test cohorts’ clinical and radiological characteristics are listed in Table 1, with their distributions shown in Table 2. There were no appreciable variations in the age, sex, history of smoking, clinical complaints, numbers of lesions, calcifications, or lesion length between the training and test cohorts. In contrast, the histopathological grading of the lesions and presence of tortuous blood vessels differed statistically throughout the training and test cohorts. Statistics showed that the differences were significant (*p* < 0.05).

**Table 1:** The training and test cohorts patients’ baseline demographics, clinical, and radiologic characteristics

Characteristics	All	Training cohort (*n* = 137)	Test cohort (*n* = 59)	p
Age, mean ± SD^a^, years	67.821 ± 10.468	68.066 ± 11.184	67.254 ± 8.64	0.360
Sex				0.662
Male	160 (81.6%)	117 (85.4%)	43 (72.9%)	
Female	36 (18.4%)	20 (14.6%)	16 (27.1%)	
Smoking				0.773
Yes	145 (73.9%)	108 (78.8%)	37 (62.7%)	
No	51 (26.1%)	38 (21.2%)	13 (37.3%)	
Clinical complaint				0.493
Hematuria	113 (57.7%)	82 (59.9%)	31 (52.5%)	
Hydronephrosis	2 (1.0%)	2 (1.5%)	0 (0%)	
Incidental finging	81 (41.3%)	53 (38.7%)	28 (47.5%)	
Number of lesions				0.141
Multiple	58 (29.6%)	45 (32.8%)	13 (22.0%)	
Single	138 (70.4%)	92 (67.2%)	46 (78.0%)	
Histopathologic grade				0.000
High grade	130 (66.3%)	92 (67.2%)	38 (64.4%)	
Low grade	66 (33.7%)	45 (32.8%)	21 (35.6%)	
Calcification				0.915
Yes	70 (35.7%)	47 (34.3%)	23 (39.0%)	
No	126 (64.3%)	90 (65.7%)	36 (61.0%)	
Lesion length, mm	17.451 ± 9.042	16.015 ± 8.215	19.015 ± 10.341	0.109
Tortuous blood vessels				0.000
Yes	81 (41.3%)	58 (42.3%)	23 (39.0%)	
No	115 (58.7%)	79 (57.7%)	36 (61.0%)	

^a^ *SD* standard deviation

**Table 2:** The distribution of clinical and radiologic characteristics in the training and test cohorts

Characteristics	Training cohort (*n* = 137)			Test cohort (*n* = 59)		
	MIBC	NMIBC	p	MIBC	NMIBC	p
Age, mean ± SD^a^, years	68.735 ± 11.043	67.406 ± 11.363	0.489	68.034 ± 7.744	66.5 ± 9.497	0.500
Sex			0.604			0.937
Male	57 (83.8%)	60 (87.0%)		21 (72.4%)	22 (73.3%)	
Female	11 (16.2%)	9 (13.0%)		8 (27.6%)	8 (26.7%)	
Smoking			0.745			0.842
Yes	51 (75%)	57 (82.6%)		15 (51.7%)	22 (73.3%)	
No	17 (25%)	12 (17.4%)		14 (48.3%)	8 (26.7%)	
Clinical complaint			0.719			0.358
Hematuria	43 (63.2%)	39 (56.5%)		17 (58.6%)	14 (46.7%)	
Hydronephrosis	1 (1.5%)	1 (1.4%)		0 (0%)	0 (0%)	
Incidental findings	24 (35.3%)	29 (42.0%)		12 (41.4%)	16 (53.3%)	
Number of lesions			0.179			0.297
Multiple	18 (26.5%)	27 (39.1%)		6 (20.7%)	7 (23.3%)	
Single	50 (73.5%)	42 (60.9%)		23 (79.3%)	23 (76.7%)	
Histopathologic grade			0.000			0.001
High grade	63 (92.6%)	29 (42.0%)		25 (86.2%)	13 (43.3%)	
Low grade	5 (7.4%)	40 (58.0%)		4 (13.8%)	17 (56.7%)	
Calcification			0.633			0.365
Yes	22 (32.4%)	25 (36.2%)		13 (44.8%)	10 (33.3%)	
No	46 (67.6%)	44 (63.8%)		16 (55.2%)	20 (66.7%)	
Lesion length, mm	17.045 ± 9.516	15.425 ± 8.341	0.191	19.851 ± 10.925	17.670 ± 9.812	0.14
Tortuous blood vessels			0.000			0.000
Yes	41 (60.3%)	17 (24.6%)		18 (62.1%)	5 (16.7%)	
No	27(39.7%)	52 (75.4%)		11 (37.9%)	25 (83.3%)	

^a^ *SD* standard deviation

### Feature selection and Radiomics score building

**Fig. 2:**
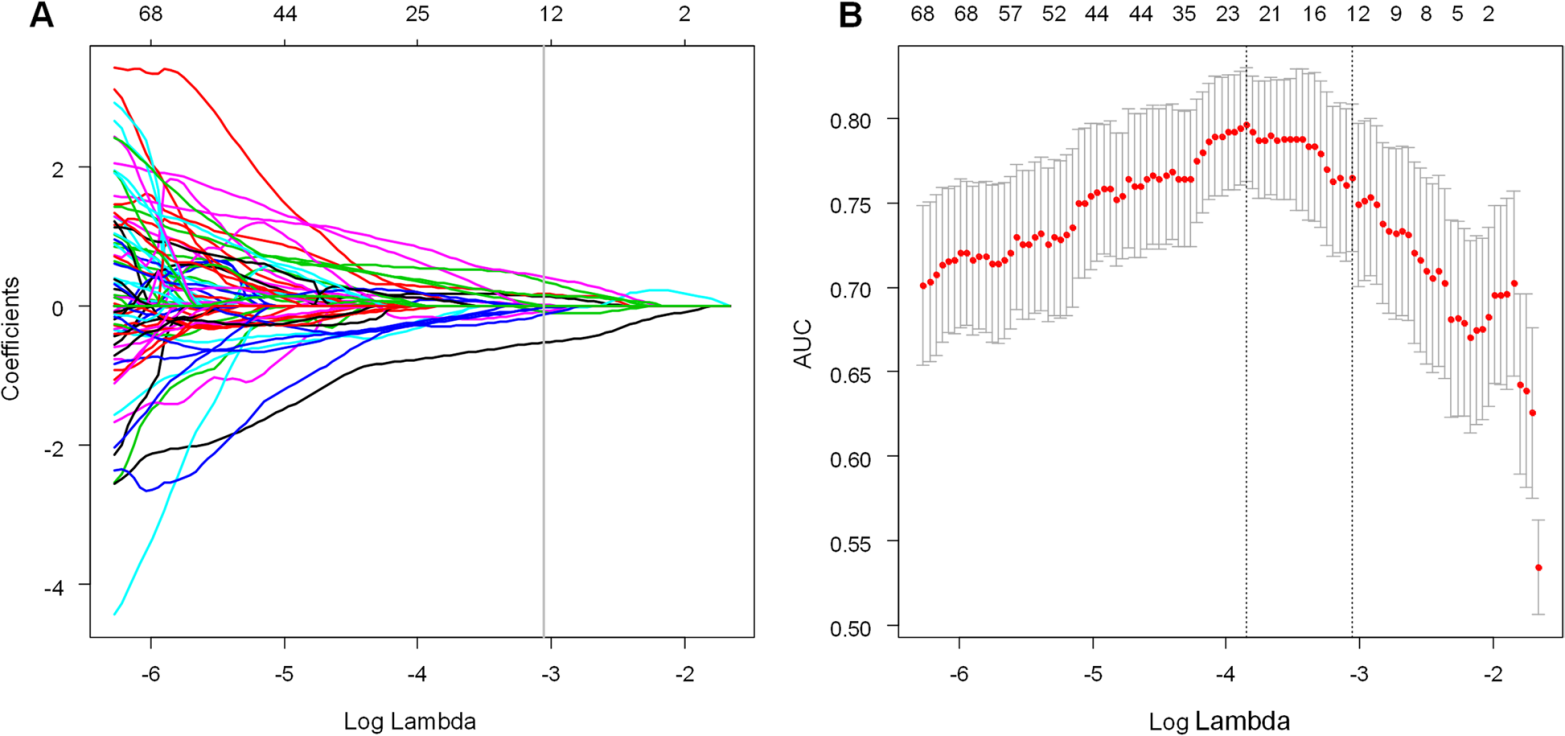
Employing the least absolute shrinkage and selection operator (LASSO) approach to select radiomics features from CT images. **A** The optimal penalty coefficient lambda (λ) for the feature of the CT images was obtained based on 10-fold cross-validation. **B** LASSO coefficient profiles of the 12 radiomics features

In total, 851 features were extracted from each lesion in the axial nephrographic-phase CT images. A total of 799 features with an ICC of more than 0.75 were extracted, including first-order, shape, gldm, glcm, glszm, glrlm, and ngtdm features. After significance test analysis, 325 features were retained. Features screening using the LASSO method with 10-fold cross-validation resulted in the selection of the 12 best radiomics features. A model was created, and these features were defined as the Radscore (Table 3). The AUC of the radiomics model in the training cohorts and test cohorts were 0.845 (95% CI, 0.781–0.908) and 0.847 (95% CI, 0.739–0.954), respectively. Figure 2 depicts the steps used in the LASSO binary logistic regression model. The following Radscore was used to establish the radiomics features:

$$\textrm{Radscore}=0.0288301363804109-0.528980248435895\ast \textrm{original}\_\textrm{shape}\_\textrm{Sphericity}-0.128902934155834\ast \textrm{wavelet}.\textrm{HHL}\_\textrm{ngtdm}\_\textrm{Strength}-0.0883729124410026\ast \textrm{wavelet}.\textrm{HHL}\_\textrm{glszm}\_\textrm{ZonePercentage}-0.078874563$$

$$\ast \textrm{wavelet}.\textrm{LHH}\_\textrm{glszm}\_\textrm{LargeAreaEmphasis}-0.027663448\ast \textrm{wavelet}.\textrm{HHH}\_\textrm{gldm}\_\textrm{SmallDependenceLowGrayLevelEmphasis}-0.019698515$$

$$\ast \textrm{wavelet}.\textrm{HHL}\_\textrm{gldm}\_\textrm{DependenceVariance}+0.011777035\ast \textrm{original}\_\textrm{gldm}\_\textrm{LargeDependenceHighGrayLevelEmphasis}+0.133742823\ast \textrm{original}\_\textrm{shape}\_\textrm{Elongation}+0.15340688\ast \textrm{wavelet}.\textrm{LLL}\_\textrm{glcm}\_\textrm{MCC}+0.169020499\ast \textrm{original}\_\textrm{shape}\_\textrm{LeastAxisLength}+0.349791508\ast \textrm{wavelet}.\textrm{HLL}\_\textrm{glcm}\_\textrm{Correlation}+0.413107304\ast \textrm{wavelet}.\textrm{HLH}\_\textrm{ngtdm}\_\textrm{Busyness}$$

**Table 3:** Twelve radiomics features and their correlation coefficients

Correlation coefficient	Features
−0.52898	original_shape_Sphericity
−0.1289	wavelet.HHL_ngtdm_Strength
−0.08837	wavelet.HHL_glszm_ZonePercentage
−0.07887	wavelet.LHH_glszm_LargeAreaEmphasis
−0.02766	wavelet.HHH_gldm_SmallDependenceLowGrayLevelEmphasis
−0.0197	wavelet.HHL_gldm_DependenceVariance
0.011777	original_gldm_LargeDependenceHighGrayLevelEmphasis
0.133743	original_shape_Elongation
0.153407	wavelet.LLL_glcm_MCC
0.16902	original_shape_LeastAxisLength
0.349792	wavelet.HLL_glcm_Correlation
0.413107	wavelet.HLH_ngtdm_Busyness

### Development and performance of a Radiomics-clinical nomogram

The results of univariate and multivariate analyses showed that histopathological grading and tortuous blood vessels were independent predictors for the status of muscular invasion in bladder cancer (Table 4). Based on a few chosen clinical indicators, the clinical model was created. In the training cohorts, the clinical model’s AUC values and 95% CI, sensitivity, and specificity were 0.811 (95% CI, 0.742–0.880), 0.926, and 0.579 while in the test cohorts, they were 0.808 (95% CI, 0.703–0.913), 0.862, and 0.567 (Table 5).

**Table 4:** Clinical predictors of BCa: Univariate and Multivariate Analyses

Variables	Univariate analyses		Multivariate analyses		Multivariate analyses	
	OR(95% CI)	*p* value	OR(95% CI)	*p* value	OR(95% CI)	*p* value
Age	1.002 (0.968–1.037)	0.352				
Sex	1.278 (0.528–3.092)	0.664				
Smoking	1.513 (0.696–5.184)	0.773				
Clinical complaint	1.094 (0.518–2.310)	0.237				
Number of lesions	0.483 (0.225–1.036)	0.142				
Histopathologic grade	12.058 (5.133–28.325)	0.000	13.803 (4.832–39.429)	0.000	10.787 (3.438–33.842)	0.000
Calcification	0.687 (0.326–1.447)	0.916				
Lesion length	0.348 (0.225–1.152)	0.109				
Tortuous blood vessels	4.289 (2.064–8.912)	0.000	3.097 (1.346–7.125)	0.007	2.375 (0.752–5.049)	0.084
Radscore					6.554 (2.986–14.387)	0.000

**Table 5:** Performance of clinical, radiomic models and radiomics-clinical nomogram

	AUC*(95%CI)	Sensitivity(95%CI)	Specificity(95%CI)	Accuracy(95%CI)	NPV**(95%CI)	PPV***(95%CI)
Train						
Radiomic	0.845 (0.781–0.908)	0.705 (0.585–0.808)	0.826 (0.739–0.913)	0.766 (0.686–0.834)	0.740 (0.642–0.838)	0.800 (0.698–0.901)
Clinical model	0.811 (0.742–0.880)	0.926 (0.867–0.985)	0.579 (0.464–0.695)	0.752 (0.671–0.822)	0.888 (0.797–0.890)	0.685 (0.590–0.779)
Radiomics-clinical nomogram	0.896 (0.846–0.947)	0.867 (0.794–0.941)	0.797 (0.696–0.884)	0.832 (0.759–0.890)	0.859 (0.774–0.944)	0.808 (0.717–0.898)
Test						
Radiomic	0.847 (0.739–0.954)	0.706 (0.588–0.809)	0.766 (0.6–0.9)	0.779 (0.653–0.877)	0.793 (0.645–0.940)	0.767 (0.615–0.918)
Clinical model	0.808 (0.703–0.913)	0.862 (0.724–0.965)	0.567 (0.367–0.733)	0.712 (0.579–0.822)	0.809 (0.641–0.977)	0.657 (0.507–0.809)
Radiomics-clinical nomogram	0.887 (0.803–0.971)	0.793 (0.655–0.931)	0.733 (0.566–0.867)	0.763 (0.634–0.864)	0.786 (0.634–0.938)	0.742 (0.587–0.895)

*AUC area under the curve, **PPV positive predictive value, ***NPV negative predictive value

**Fig. 3:**
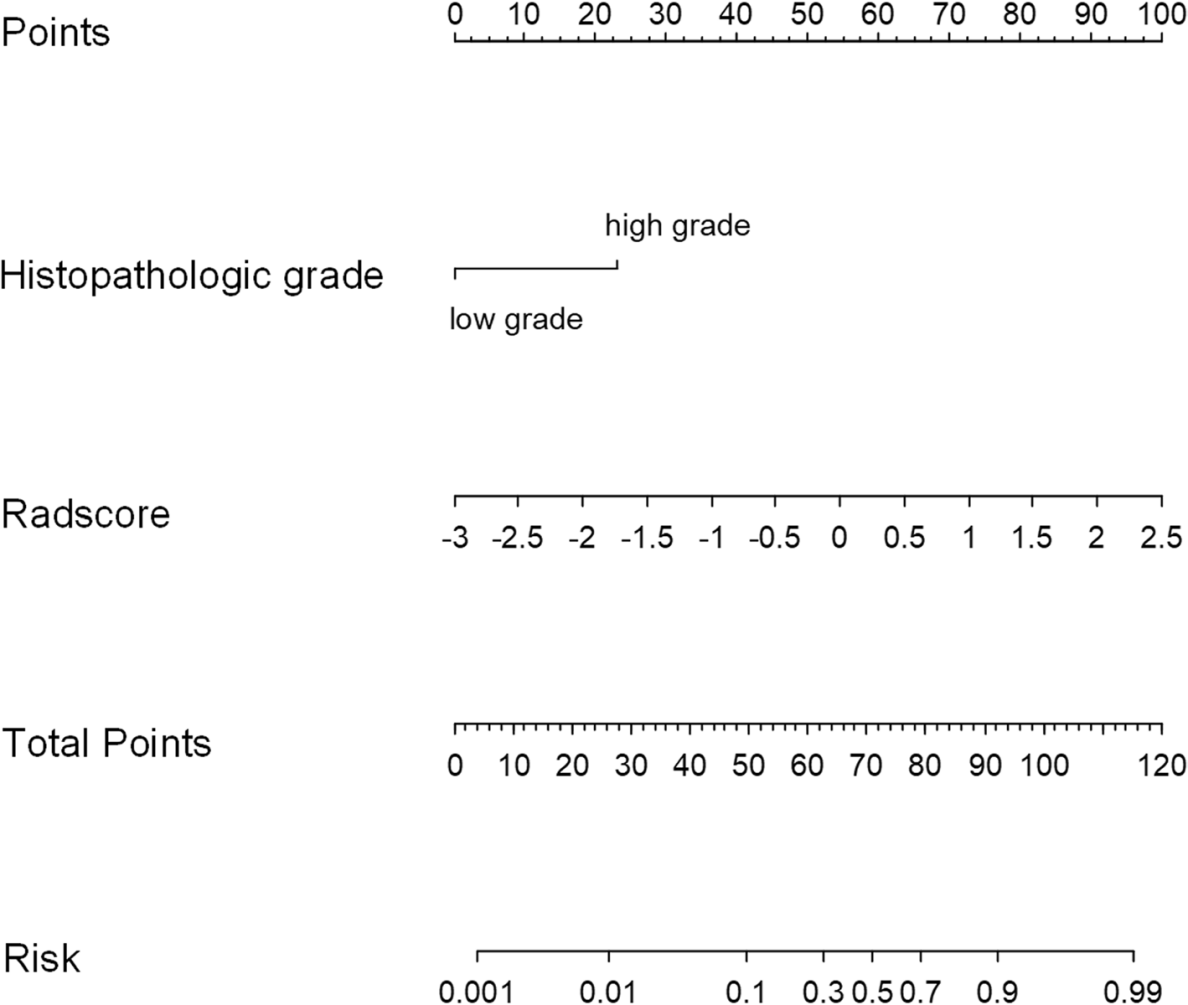
By combining the Radscore with histopathological grading, the radiomics-clinical nomogram was created

**Fig. 4:**
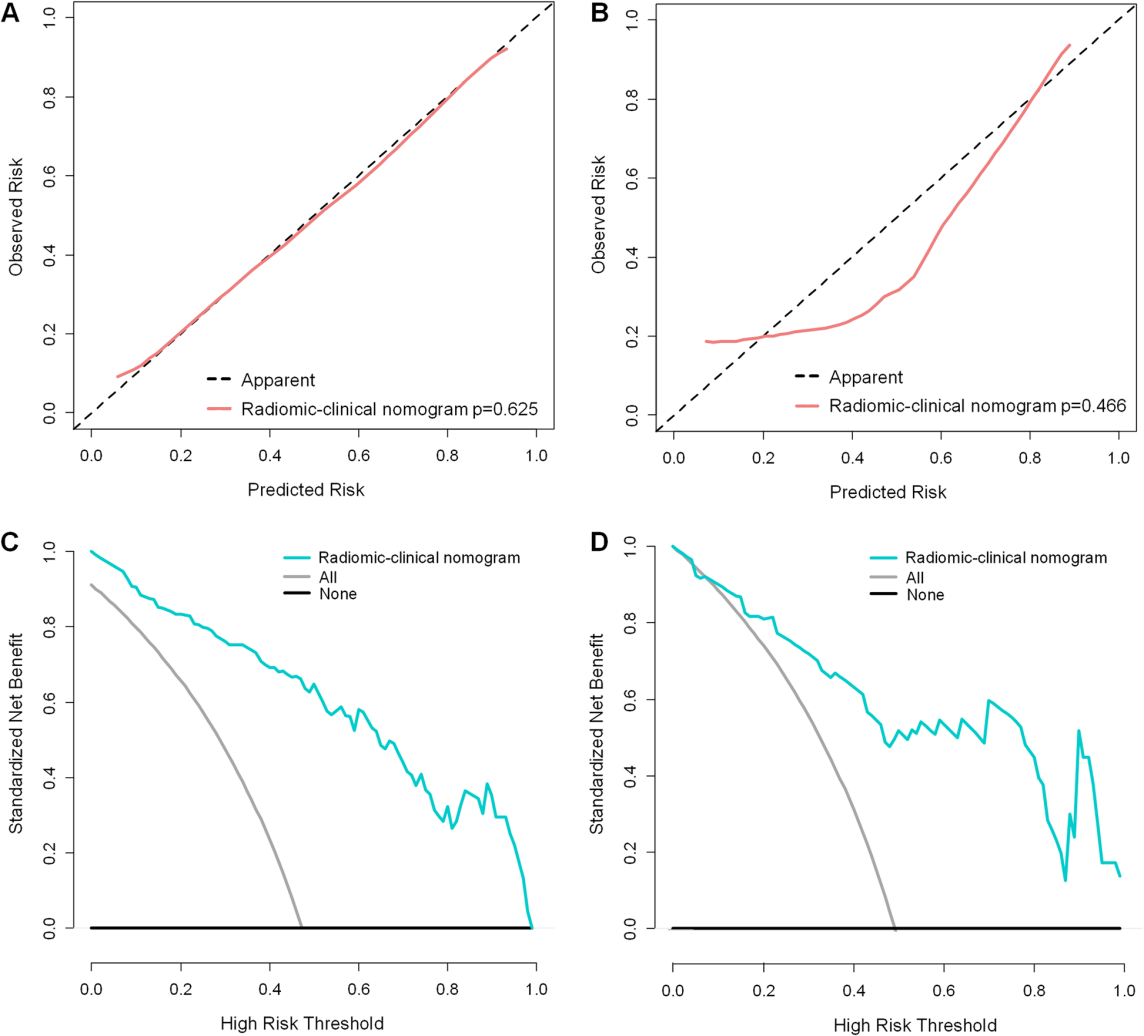
Calibration curves of the radiomics-clinical nomogram in the training (**A**) and test cohorts (**B**). Decision curve analyses (DCA) for the combined model in the training (**C**) and test cohorts (**D**)

Based on the clinical indicators in the clinical model, multifactorial analysis was then performed in conjunction with the obtained Radscore. Details are provided in Table 4. The two indicators clinical histopathological grading and Radscore were the final predictors. A radiomics-clinical nomogram was created based on the selected predictors, as shown in Fig. 3. The calibration curve showed that both the training and test cohorts’ predictions and observations had a high degree of agreement (Fig. 4). With respect to the radiomic-clinical nomogram, the Hosmer-Lemeshow test produced non-significant *p* values in both cohorts (*p* = 0.625 and 0.466, respectively), indicating adequate calibration.

### Models comparison and clinical usefulness evaluation

**Fig. 5:**
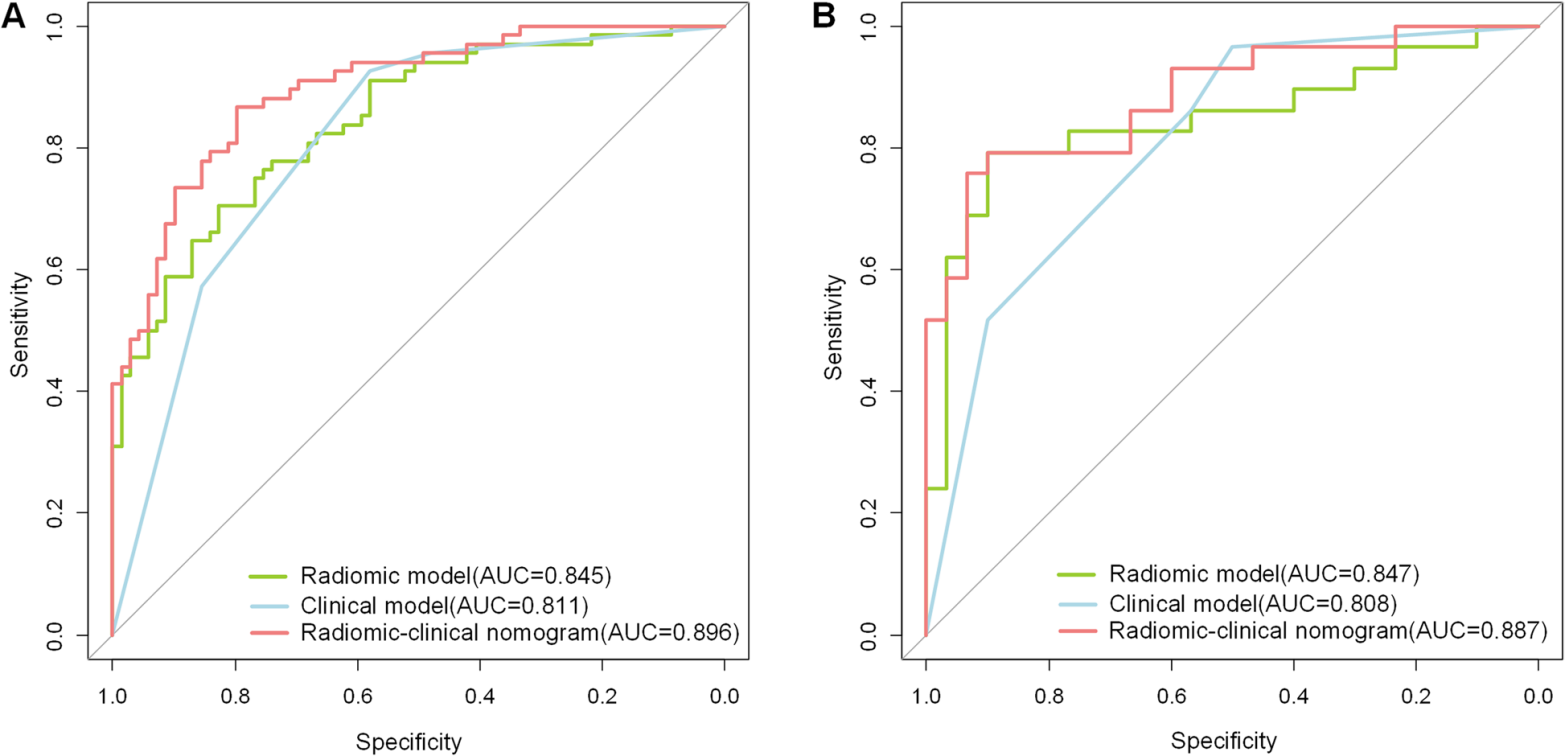
The clinical, radiomics, and radiomics-clinical nomogram receiver operating characteristic (ROC) curves. The ROC curve shows the radiomics-clinical nomogram is better than the separate CT-based radiomics model and the clinical model in the training (**A**) and test (**B**) cohorts

Figure 3 displays the ROC curves for the three models. Details about the model’s diagnostic performance in the training and test cohorts are included in Table 2. A comparison of the three models’ ROC curves is shown in Fig. 5. The radiomics-clinical nomogram had good predictive ability, with AUC values of 0.896 (95% CI, 0.846–0.947) in the training cohorts and 0.887 (95% CI, 0.803–0.971) in the test cohorts, which were slightly better than the AUC values of the radiomics model. Finally, the diagnostic capabilities of the three models were evaluated using the DeLong test, as shown in Table 6. In the training cohort, the DeLong’s test displayed significant difference in AUC between the radiomics-clinical nomogram and the radiomics model (0.896 versus 0.845, *p* = 0.015). The radiomics-clinical nomogram compared with the clinical model (0.896 versus 0.811) for discriminating muscle invasive in the training cohort demonstrated significant outcomes with the *p* value of 0.002. Delong tests in the test cohort revealed that there was no significant difference among the three models (nomogram versus radiomics, *p* = 0.185; nomogram versus clinical, *p* = 0.098). The decision curve for the radiomics-clinical nomogram is depicted in Fig. 5.

**Table 6:** Comparison of the AUC Values among models

	Training cohort			Test cohort		
		*p *Value in Comparison to radiomic model	*p* Value in Comparison to clinical model		p Value in Comparison to radiomic model	p Value in Comparison to clinical model
Model	^a^AUC(95% CIs)			AUC(95% CIs)		
Radiomic	0.845 (0.781–0.908)		0.415	0.847 (0.739–0.954)		0.567
Clinical	0.811 (0.742–0.880)	0.415		0.808 (0.703–0.913)	0.567	
Radiomics-clinical nomogram	0.896 (0.846–0.947)	0.015	0.002	0.887 (0.803–0.971)	0.185	0.098

^*^AUC area under the curve, p < 0.05, which is considered statistically significant difference

## Discussion

In the study, we successfully constructed a radiomics-clinical nomogram for preoperative prediction of muscle invasion status in BCa. By comparing the AUC values derived from the ROC curve analysis, the radiomics-clinical nomogram was superior to the other two models (clinical and radiomics) in the training and test cohorts. The same pattern was seen in terms of accuracy and specificity. Finally, the results of the DeLong test demonstrate that the radiomics-clinical nomogram in the training cohort had the best diagnostic performance. The nomogram combining CT radiographic features and clinical risk factors showed better discriminatory and predictive power than the radiographic models in distinguishing MIBC from NMIBC.

The CT examination is time-efficient and has a good sensitivity for detecting calcifications. It provides a visual representation of the bladder wall’s structure, any changes in thickness, and the involvement of adjacent tissues after augmentation [7, 11, 12]. This study included only individuals who had undergone TURBT or cystectomy and had BCA muscle infiltration confirmed. Notably, TURBT pathologic specimens included the muscular layer of the bladder wall, which is characteristic for NMIBC cases [10]. This study examined multiple clinical data points before arriving at statistically significant conclusions. In the clinical model of this study, we included tortuous vessels and histopathological grading. BCas are blood-rich tumors that require nutrient supply for growth, resulting in neoplastic angiogenesis within the lesions [13, 14]. Tumor growth depends on neovascularization within the tumor [14]. Tortuous blood-supplying vessels may be detected in CT-enhanced images of larger tumor lesions [13, 15]. However, the clinical indicator of tortuous vessels was not included in the final radiomics-clinical nomogram after *p* = 0.084 was found in the multifactorial analysis. It is possible that the tortuous vessel in the CT imaging is a subjective judgment of the radiologist, so it needs to be supported by more cases or multicenter cases.

Clinical staging may commonly be affected by cystoscopic or imaging manifestations, multiple biopsies [16]. This study’s model incorporated pathological tissue grading of the biopsy [17]. Because of the influence of biopsy sampling site, the size of the tissue sampled, and other factors, preoperative and postoperative pathological findings may differ slightly between [5, 18]. Preoperative tissue grading, on the other hand, is useful in predicting the status of BCa muscle layer infiltration. Almost half of newly diagnosed NMIBC are low grade, while the vast majority of MIBC are high-grade [17, 19, 20]. This is consistent with what we discovered. However, it has also been reported in the literature that one-third of NMIBC consist of high-grade BCas [21]. Pathological phenotypes such as grading, staging, and muscle invasion status, according to European Association of Urology (EAU) guidelines, have a significant impact on treatment decisions and prognosis [19, 20]. Li et al. developed a radiomics model for preoperative assessment of histological demarcation of NMIBC based on clinical and radiological data from 169 NMIBC patients [6]. In their study, age and tumor number were included in the model as clinical factors. We also collected age and number of tumors as clinical baseline information, but these features were not finally included in the model.

Radiomics is a technique for extracting and analyzing quantitative imaging features from medical images (e.g., CT, MRI, and other images) [22]. Radiomics is used to develop descriptive and predictive models [14]. In this study, 12 features included morphological features and wavelet filtered features were ultimately extracted from each BCa by radiomics analysis to create radiomics model for distinguishing the infiltration status of bladder intrinsic muscle.

Morphological features included original_shape_Sphericity, original_shape_Elongation, original_shape_LeastAxisLength. The likelihood of bladder cancer muscle invasion was higher when the tumor shape was close to a spherical ellipse. Some scholars predicted the muscle invasion status of bladder cancer based on the tumor-bladder wall contact length (TCL) [23]. Qing Li et al. [23] discovered that an elevated TCL was independently correlated with the muscle infiltration of bladder cancer. Akcay et al. also found that the simultaneous use of VI-RADS criteria and TCL in mpMRI to assess muscular invasion in BCa patients was a successful and highly reproducible method [24]. Tumor size has prognostic and predictive significance in NMIBC, according to EAU guidelines [25]. In this study, an original feature named original_shape_LeastAxisLength was included in the radiomics features. We also included the transverse axial lesion length in CTU images and revealed that the tumor lesion length in MIBC surpassed that of NMIBC. Nevertheless, the difference observed in the training and test groups did not attain statistical significance. This may suggest the superiority of lesion size-related features in radiomics over conventional imaging measurements. The radiomics features contained more wavelet filtered features. The wavelet transform can also gradually convert image information into low- and high-frequency information, which improves local features, increases information content in tumor images, and provides more information about the biological behaviors and heterogeneity of different tumors at multiple scales [26].

The nomogram is a great illustration of how several pertinent pieces of information can be combined to predict a specific endpoint by showcasing the outcomes of a predictive model on a scale. The nomogram can be used to visually determine the patient’s risk for the matching MIBC on the prediction line at the bottom of the nomogram, which can be used to inform the doctor’s decisions about the best course of therapy. The nomogram produced by combining the Radscore and clinical data for the test cohort had an AUC of 0.887, suggesting that the constructed model had sufficient MIBC prediction accuracy. Consequently, the combined nomogram prediction model may aid in directing physicians’ choices.

Our study have several limitations. First, the sample size is quite modest. Second, there is a selection bias, which can restrict the proposed model’s applicability. This study excluded some ineligible cases, for example cases with a small lesion size or the absence of muscle components in the pathological tissue. Additionally, the radiomics model was not externally validated in this study. It is necessary to perform a multicenter validation with more participants.

In summary, to assess muscle invasion in BCa prior surgery, a radiomics-clinical nomogram based on CT images was built in this study. The nomogram showed a high level of diagnostic efficacy and can be used as a guide for BCa prognostic evaluation and tailored treatment.

### Supplementary Information

**Supplementary Material 1.**

## Data Availability

The datasets generated during the current study are included in this published article. Further inquiries can be available from the corresponding author.
