# Supplementary material for: Predicting preoperative muscle invasion status for bladder cancer using computed tomography-based radiomics nomogram
Source: BMC Med Imaging. 2024 Apr 27;24:98. doi: 10.1186/s12880-024-01276-7 (PMC11055285; doi:10.1186/s12880-024-01276-7)
Supplement: Supplementary file 1 — Supplementary Material 1. [file 12880_2024_1276_MOESM1_ESM.docx]

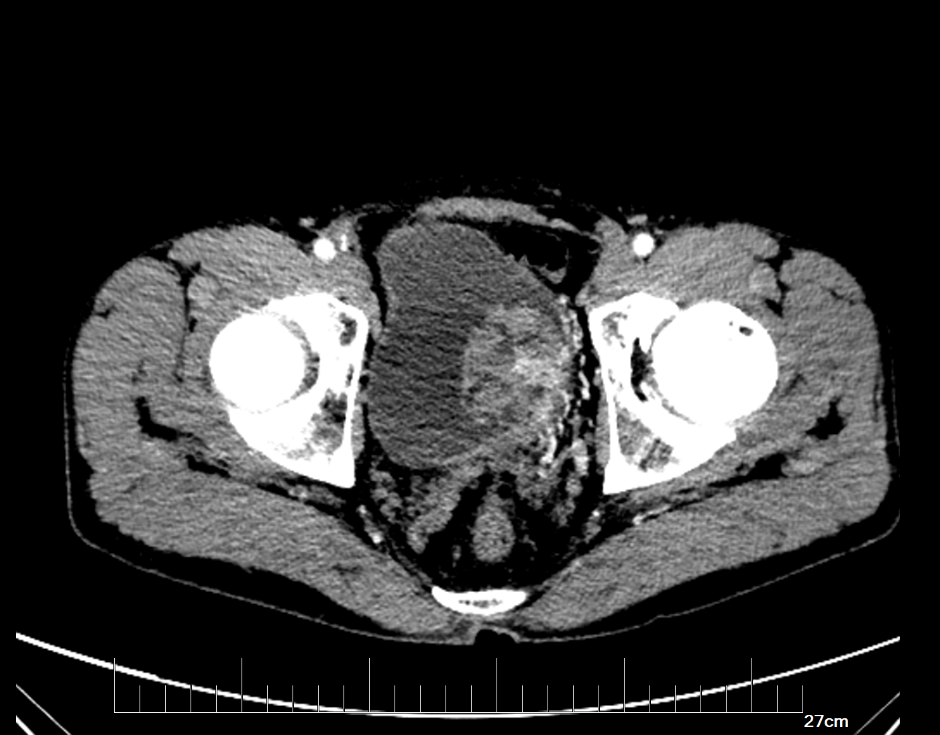


Multiple tortuous vascular are seen within and around the lesion on CTU images in patients with high-grade invasive bladder cancer.
